# *Selaginella guihaia* (Selaginellaceae): A new spikemoss species from southern China and northern Vietnam around the Gulf of Tonkin

**DOI:** 10.3897/phytokeys.80.11126

**Published:** 2017-05-17

**Authors:** Yu-Dong Wu, Hong-Rui Zhang, Xian-Chun Zhang

**Affiliations:** 1 State Key Laboratory of Systematic and Evolutionary Botany, Institute of Botany, Chinese Academy of Sciences, Beijing, 100093, China; 2 University of Chinese Academy of Sciences, Beijing 100049, China

**Keywords:** Lycopodiophyta, lycophytes, taxonomy, new species, *rbcL*, ITS

## Abstract

*Selaginella guihaia* **sp. nov.** (Selaginellaceae), a new species of spikemoss from southern China and northern Vietnam around the Gulf of Tonkin (Beibu Gulf), is described and illustrated. Morphological and molecular comparisons of the new species with other similar species (*S. doederleinii*, *S. ornata* and *S. trachyphylla*) are provided. The morphological and molecular evidence clearly indicates *S. guihaia* is a distinct species. Morphologically *S. guihaia* differs from other species by its obviously white–margined leaves, the ventral leaves scabrous on upper surfaces throughout the basiscopic or also rarely present on upper halves, and the ovate axillary leaves.

## Introduction

*Selaginella* P. Beauv. (Selaginellaceae) is the largest lycophyte genus with about 700–800 species and distributed in all the continents except Antarctica (Jermy 1990; Tryon and Lugardon 1991; Zhang 2004; Zhang et al. 2013; Zhou and Zhang 2015; Zhou et al. 2016; Weststrand and Korall 2016a, b; PPG I 2016). However, the highest species diversity occurs in the tropics and subtropics. The genus is characterized by the presence of rhizophores, single veined leaves with ligule, sporangia borne axillary on the upper surface of sporophylls and bearing two types of spores (heterospory) (Webster 1992). Among several herbaria collections of *Selaginella doederleinii* Hieron., we found that the leaves of some specimens are obvious white–margined with ventral leaves that are often scabrous on the upper surface. We also observed and collected similar plants in the field. These turned out to be a rather common and widely distributed undescribed species in the mountainous areas of southern China (Guangxi and Hainan) and North Vietnam around the Gulf of Tonkin (Beibu Gulf). With evidences from morphological characters and molecular analysis, we described these plants as a new species herein.

## Materials and methods

Morphology characters were examined from the dried herbarium specimens studied from PE (herbaria acronyms according to Thiers 2016). All the characters were examined under stereomicroscope using NIS‐Elements D 3.10 imaging software from Nikon Instruments (http://www.nikoninstruments.com). Voucher specimens (see Appendix 1) are deposited at PE.

We downloaded 60 sequences ITS and *rbcL* from Genbank representing 32 species in *Selaginella* and those species involves the major clades of the phylogenetic analysis of *Selaginella* (Zhou et al. 2016). In this study, we newly sequenced four species, including two samples of the possible new taxon and four samples of its putative relatives, *S. ornata* (Hook. & Grev.) Spring, *S. doederleinii* Hieron. and *S. trachyphylla* A. Braun. Total genomic DNA was isolated from silica–dried material using the Plant Genomic DNA Kit (Tiangen Biotech, Beijing, China) following the manufacturer’s protocols. One plastid region *rbcL* and one nuclear region ITS were amplified for the possible new taxon and its putative closely related taxa. The *rbcL* region was amplified with newly designed primers *rbcL* 192F (5’ CACGTGGACTACCGTTTGGA3’) and 1324R (TACCCTCAAGAGCGGGATCA3’). The primers were designed in Primer 3.0 (Untergasser et al. 2012) using the published chloroplast genomes of *Selaginella moellendorffii* Hieron. (Smith 2009) and *S. uncinata* (Desv.) Spring (Tsuji et al. 2007). The PCR protocol of *rbcL* region followed Zhou et al. (2016). The ITS region was amplified using the primers and PCR protocol described in Arrigo et al. (2013). All PCR products were directly sequenced using ABI 3730XL analyzer (Applied Biosystems, Foster City, California, USA). Newly obtained sequences were assembled with ContigExpress and then aligned with the downloaded sequences using Clustal X v.1.83 (Thompson et al. 1997) followed by manual adjustment in BioEdit v.7.1.11(Hall 1999). The full length of the ITS region were sequenced but only 5.8S and part of ITS2 region were used because of a large number of insertions and deletions in ITS1 and ITS2 (Zhou et al. 2016); the ambiguous regions were excluded prior to analysis as previously done in similar studies (Arrigo et al. 2013; Zhou et al. 2016). ILD (Incongruence Length Difference) test (Farris et al. 1995) was performed on PAUP* v.4.0b10 (Swofford 2002) to test if there is conflict between nuclear and chloroplast genes. The combined dataset (*rbcL* and ITS) were analyzed with the maximum parsimony (MP), maximum likelihood (ML) and Bayesian inference (BI) methods. MP analyses were carried out using PAUP* v.4.0b10 (Swofford 2002). All characters were weighted equally and gaps were treated as missing data. The most parsimonious trees were obtained with heuristic searches of 1000 replicates random stepwise sequence addition(RAS), with tree bisection–reconnection (TBR) branch swapping, and 10 trees from each random sequence addition were saved were used to obtain the most parsimonious trees. MP bootstrap values (MP_BS_) were calculated with 1000 replicates. jModelTest 0.1.1 (Posada 2008) was used to select the appropriate substitution model for ML and BI analyses. The ML analyses were conducted using the web server RAxML–HPC2 on XSEDE (Stamatakis 2014), and ML bootstrap values (ML_BS_) were calculated applying 1000 bootstrap replicates with the GTRCAT substitution model. Bayesian analyses and posterior probability (BI_PP_) calculation were conducted in MrBayes 3.2.6 (Ronquist et al. 2012) implemented on the CIPRES Science Gateway Portal (Miller et al. 2010). Four Markov chain Monte Carlo chains were run, each beginning with a random tree and sampling one tree every 1000 generations of 10 000 000 generations. After checking all the ESS>200 in Tracer v1.5 (Rambaut et al. 2009), the first 25% of samples were discarded as burn–in, and the remaining trees were used to calculate a 50% majority–rule consensus topology and posterior probability values.

## Results

**Figure 1.**
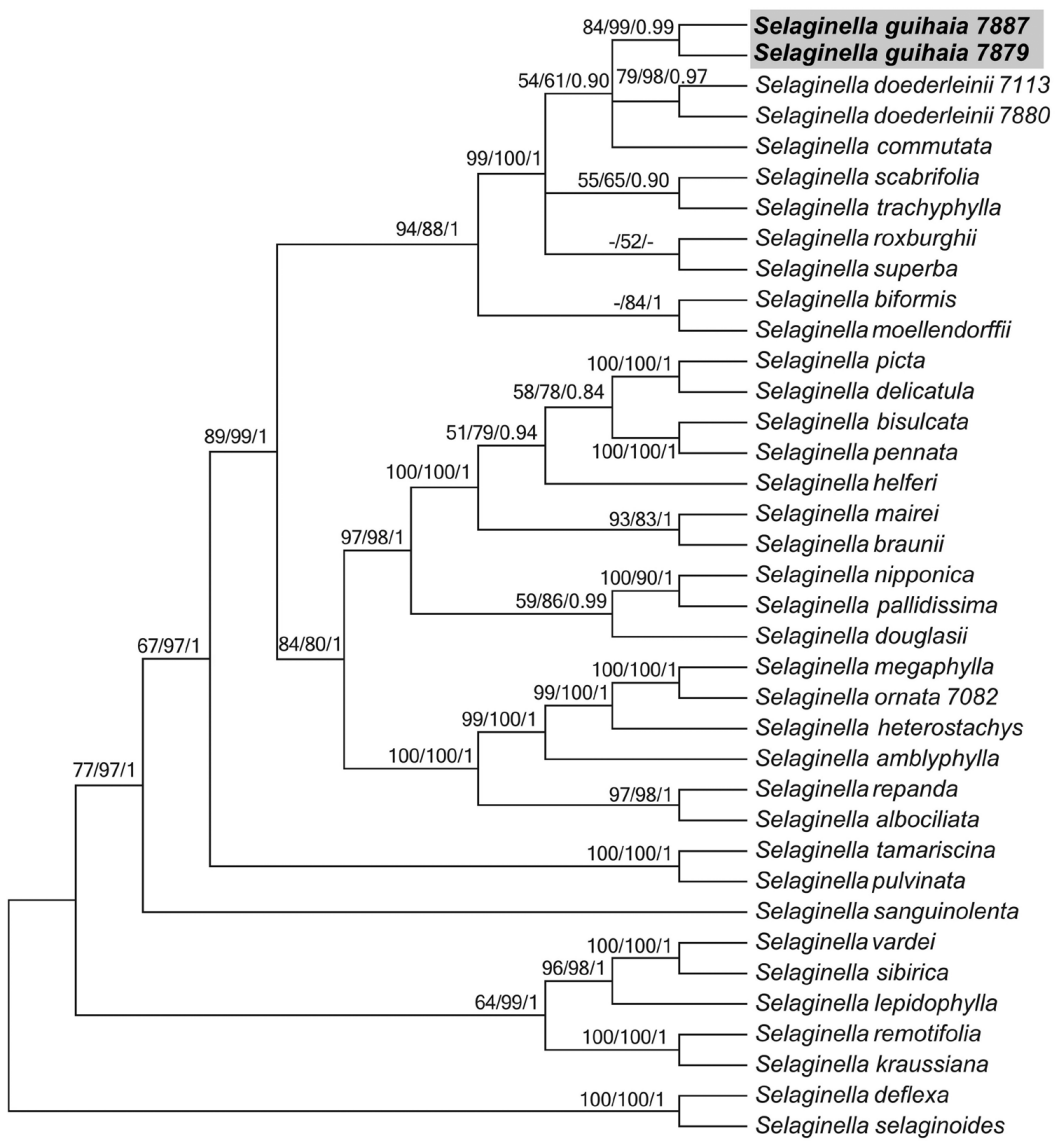
The 50% majority rule consensus tree derived from maximum likelihood showing the position of *Selaginella guihaia*. Support values (MP_BS_, ML_BS_ > 50%, BI_PP_ > 0.8) are shown above the main braches; the dash (–) indicates BS < 50%. The new species is shown in bold.

The ILD test results showed no obvious conflict existing between the two datasets, *rbcL* and ITS (P =0.02). Thus, the datasets were combined. The combined data matrix included up to 1460 nucleotides for each of the 36 taxa with 845 constant characters, 512 parsimony informative characters, consistency index (CI) = 0.56, retention index (RI) = 0.79. The three phylogenetic analyses (MP, ML, BI) inferred congruent topologies. The Best ML tree is presented in Figure 1.

The molecular evidence showed that two samples of *Selaginella guihaia* were grouped together with strong support (BS =99, PP =0.99), and then formed a moderately supported clade with *S. doederleinii* and *S. commutata* Alderw.

Our phylogenetic analyses and morphological evidence reveal that the possible new taxon *Selaginella guihaia* is different from the morphologically similar species *S. doederleinii*, *S. ornata* and *S. trachyphylla* that co-occur in the same region. The overall morphology and the growth habit of *S. guihaia* resemble those of *S. ornata*, however, the former has monomorphic (vs. dimorphic) sporophylls. Furthermore, consistent with the sporophylls variations, *S. guihaia* and *S. ornata* were separately placed into two large clades in the molecular phylogenetic tree. Although *S. doederleinii* and *S. trachyphylla* were placed closely with *S. guihaia* in the molecular phylogenetic analysis, the distinct white-margined leaves of *S. guihaia* is different from both these species. The ventral leaves of *S. guihaia* are scabrous near the lower part of leaf epidermis but rarely on the upper part, whereas hte ventral leaves of *S. trachyphylla* are scabrous throughout the leaf epidermis.

## Taxonomic treatment

### Selaginella guihaia

Taxon classificationPlantaeSelaginellalesSelaginellaceae

X.C.Zhang sp. nov.

urn:lsid:ipni.org:names:60474541-2

Figs 2 , 3 , 4

#### Diagnosis.

The new species is similar to *S. doederleinii*, *S. ornata* and *S. trachyphylla* in the habit and the morphology of dorsal leaves, ventral leaves, axillary leaves and sporophylls. However, *S. guihaia* can be easily recognized by its obvious white–margined leaves. The white-margin is about three cells wide in *S. guihaia*, but it is only one cell wide in *S. doederleinii*, *S. ornata*, and *S. trachyphylla*.

**Figure 2.**
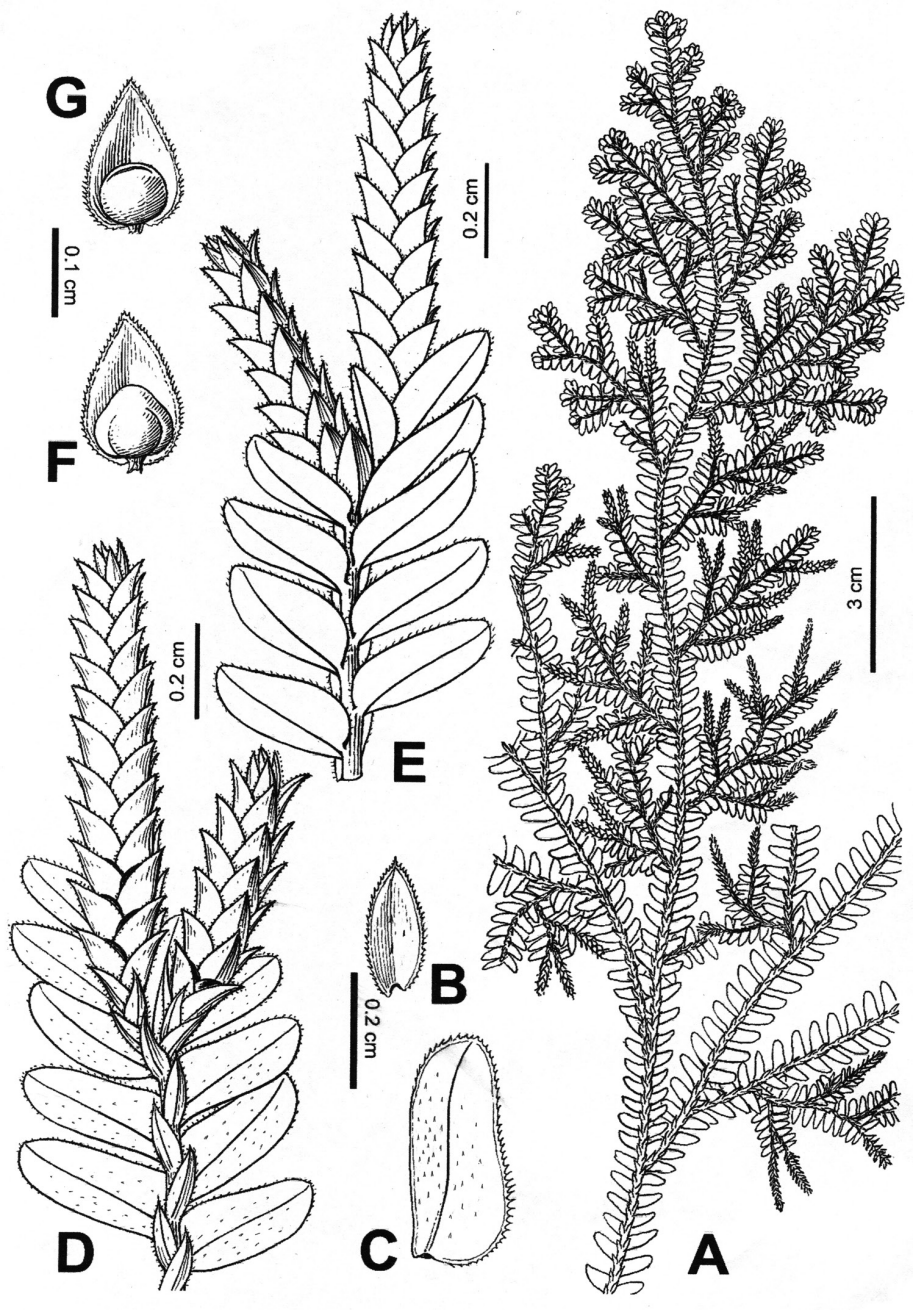
*Selaginella guihaia* X.C.Zhang **A** Habit **B** Dorsal leaf **C** Ventral leaf **D** Part of main stem showing ventral leaves, dorsal leaves, and strobili **E** Part of main stem showing ventral leaves, axillary leaves, and strobili **F** Megasporophyll **G** Microsporophyll (Drawn by C.Z. Ji from *Beijing Youth Expedition 0980*, PE).

#### Type.

**Figure 3.**
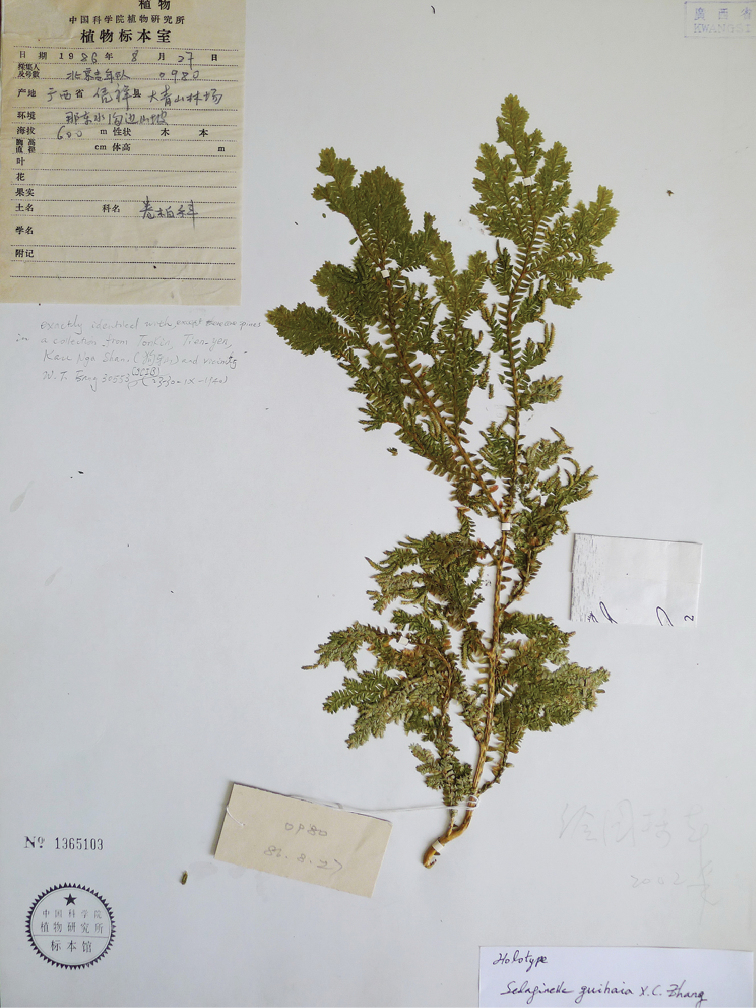
Type of *Selaginella guihaia* X.C.Zhang, sp. nov. (PE).

China, Guangxi: Pingxiang, Mt. Daqingshan, alt. 600m, 27 Aug 1986, *Beijing Youth Expedition 0980* (Holotype: PE![No. 1365103]) (Figure 3).

#### Description.

*Terrestrial*. Evergreen, suberect or ascending from decumbent base, 20–50 cm. *Rhizophores* branched from base to middle of main stem. *Main stems* pinnately branched from lower part upward, stramineous, 1.5–2 mm in diam. in lower part, oval or subquadrangular, glabrous; primary leafy branches 3–10 pairs, 2 or 3 pinnately branched, secondary branches once pinnately branched, tertiary branches forked, branchlets sparse, adjacent primary branches on main stem 1–6 cm apart, leafy portion of main stem including leaves 0.8–1.8 mm wide at middle, ultimate branches 3–6 mm wide including leaves. *Axillary leaves* on branches symmetrical, ovate, 0.9–1.7 × 1.7–3.7 mm, bases exauriculate, margins denticulate, obviously white–margined. *Dorsal leaves* on branches imbricate, ovate, 0.9–2.3 × 0.3–0.9 mm, carinate, base cuneate or obliquely subcordate, margins denticulate, obviously white–margined, apices acuminate to aristate, parallel to axis. *Ventral leaves* on branches contiguous or overlapping, slightly ascending, oblong–falcate, 2.1–4.4 × 0.8–1.9 mm, upper surfaces on lower scabrous halves of the laminae or also rarely scabrous on upper half; basiscopic base margin entire, margin subentire, denticulate at base; acroscopic base rounded, overlapping stem and branches, margin denticulate in basal half, obviously white–margined. *Strobili* solitary or in pairs, terminal, compact, tetragonal, 0.8–1.4 × 0.4–0.8 mm; sporophylls monomorphic, ovate–triangular, carinate, margins denticulate, obviously white–margined, apices acuminate; sporangia pale yellow to pale brown; megasporangia spherical; microsporangia elliptic–oblong, relatively thick, marginal cells differentiated; megaspores whitish, microspores pale yellow.

#### Specimens examined.

**China. Guangxi**: Ningming, 31 Dec 2015, *X.C.Zhang & al.7879* (PE); Ningming, 1 Jan 2016, *X.C.Zhang & al. 7886* (PE), *X.C.Zhang & al. 7887* (PE), *X.C.Zhang & al. 7899* (PE), *X.C.Zhang & al. 7900* (PE); Shangsi, 17 Sep 2009, Mt. Shiwandashan, 600 m, *R.H.Jiang 059* (PE); Shangsi, Mt. Shiwandasahn 10 Jun 2009, 380m, *S.Y. Dong 2932* (IBSC); Fangchenggang, Fulong Village, Mt. Pinglong, 360 m, 19 Sep 2009, *R.H. Jiang 145* (PE); Fangchenggang, Nale Village, 250 m, 20 Sep 2009, *R.H.Jiang 174* (PE); Shangsi, 22 Sep 2009, Mt. Shiwandashan, 680 m, *R.H.Jiang 220* (PE); Fusui, Lucheng, 200–370 m, 26 Apr 1957, *S.Q. Chen 12074* (PE); Shang–sze (= Shangsi), Shap Man Taai Shan (= Mt. Shiwandashan), 11–30 Jul 1934, *W.T.Tsang 23870* (BM); **Hainan**: Baisha, Yinggeling, 1000 m, 27 Aug 2005, *S.Y.Dong 1450* 1464 (PE). **Vietnam**: Tien–yen, Kau Nga Shan and Vicinity, 23–30 Sep 1940, *W.T.Tsang 30553* (PE); Chuk–phai, Ha–coi, Taai Wong Mo Shan and Vicinity, *W.T.Tsang 29052* (P); Dam–ha, Sai wong Mo Shan, 18 Jul – 9 Sep 1940, *W.T.Tsang 30273* (P); Tien–yen, Kau Nga Shan and Vicinity, 23 Sep – 7 Oct. 1940, *W.T.Tsang 30582* (P); Chuk–phai, Ha–coi, Taai Wong Mo Shan and Vicinity, 18 Nov – 2 Dec 1936, *W.T.Tsang 27196* (P); Tonkin, Kau Nga Shan and vicinity, Sept. 23 – Oct. 7. 1940, *W.T.Tsang 30582* (B).

#### Distribution and ecology.

**Figure 4.**
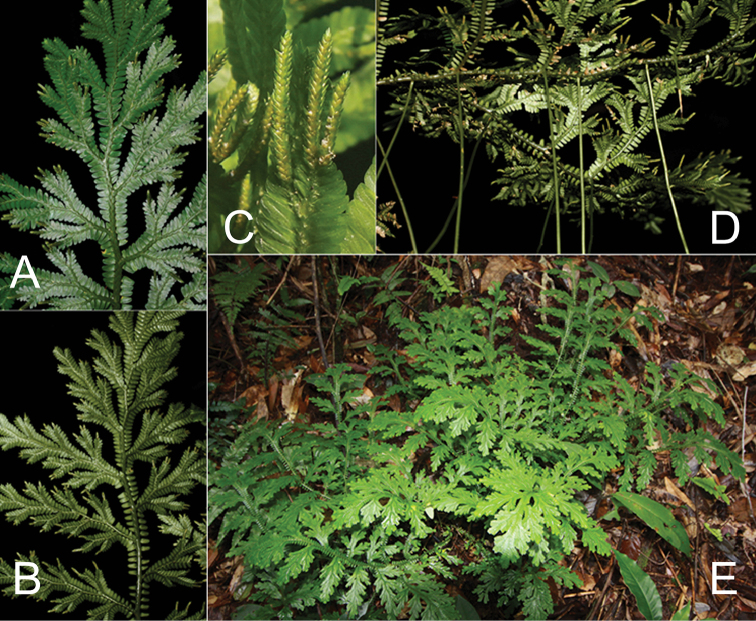
*Selaginella guihaia* X.C.Zhang, sp. nov. **A** Dorsal view of branch **B** Ventral view of branch. **C** Strobilus **D** Rhizophore **E** Habit.

**Figure 5.**
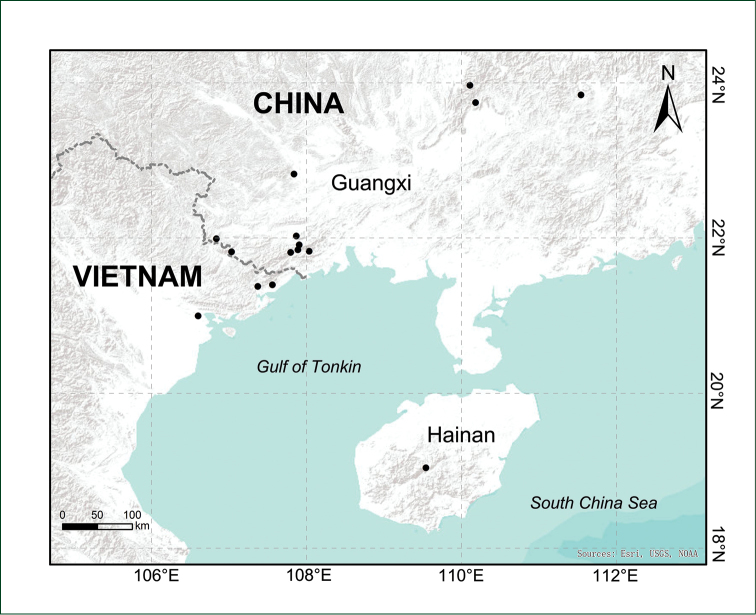
Distribution map of *Selaginella guihaia* X.C.Zhang, sp. nov.

Widely distributed in southern China (Guangxi and Hainan) and northern Vietnam around the Gulf of Tonkin (Beibu Gulf), growing in evergreen broad–leaved forests at 250 to 1000 m a.s.l. (Figures 4, 5).

#### Etymology.

The specific epithet “*guihaia*” alludes to the ancient Chinese name for the remote geographic region where the species occurs.

#### Conservation status.

We evaluated the conservation status of *Selaginella guihaia* according to the IUCN (2012) criteria for risk assessment: *S. guihaia* falls into the Least Concern (LC) category. *S. guihaia* is in fact known from many localities from southern China and northern Vietnam around the Gulf of Tonkin.

### Key to *Selaginella guihaia* and similar species

**Table:** 

1	Sporophylls dimorphic, dorsal sporophylls longer than ventral ones; megaspores reddish brown	***S. ornata***
–	Sporophylls monomorphic, dorsal sporophylls the same length as the ventral ones; megaspores whitish
2	Ventral leaf upper surfaces glabrous; basiscopic base slightly dilated; microspores yellow-orange	***S. doederleinii***
–	Ventral leaf upper surfaces scabrous; basiscopic base entire; microspore pale yellow
3	Leaves with obvious white margin (three cells wide); ventral leaves scabrous on upper surfaces only on the basiscopic halves (rarely also on upper halves); axillary leaves ovate	***S. guihaia***
–	Leaves without obvious white margin (only one cell wide); ventral leaves scabrous on upper surfaces throughout the leaf surface; axillary leaves narrowly ovate to triangular	***S. trachyphylla***

## Supplementary Material

XML Treatment for Selaginella guihaia

## Appendix

Plant materials used in this study. Information is presented in the following order: taxon name, locality (if available), collection number (if available), ITS GenBank accession number, *rbcL* GenBank accession number, references (if available). –, sequences not available. *, sequences obtained in this study.

***Selaginella albociliata*** P. S. Wang, KT161648, KT161379. ***Selaginella amblyphylla*** Alston, KT161650, KT161381. ***Selaginella biformis*** A. Braun ex Kuhn, KT161664, KT161396. ***Selaginella bisulcata*** Spring, KT161674, KT161406. ***Selaginella braunii*** Baker, KT161685, KT161419. ***Selaginella commutata*** Alderw., KT161693, KT161430. ***Selaginella deflexa*** Brackenridge, AF418999, AF093253. ***Selaginella delicatula*** (Desv.) Alston, KT161699, KT161441. ***Selaginella doederleinii*** Hieron., CHINA, Guizhou, *X.–C. Zhang et al. 7113* (PE), KY06835*, KY068356*. *X.–C. Zhang et al. 7880* (PE), KY068357*, KY068352*. ***Selaginella douglasii*** (Hook. & Grev.) Spring, –, AF419049. ***Selaginella guihaia*** X.C. Zhang, CHINA, Guangxi, *X.–C. Zhang 7879* (PE), KY068358*, KY068353*. *X.–C. Zhang 7887* (PE), CHINA, Guangxi, KY068359*, KY068354*. ***Selaginella helferi*** Warb., KT161723, KT161470. ***Selaginella heterostachys*** Baker, KT161729, KT161480. ***Selaginella kraussiana*** A. Braun. KT161746, KT161498. ***Selaginella lepidophylla*** (Hook. & Grev.) Spring, AF419002, AF419051. ***Selaginella mairei*** Levl., KT161764, KT161518. ***Selaginella megaphylla*** Baker, KT161768, KT161524. ***Selaginella moellendorffii*** Hieron., KT161777, KT161531. ***Selaginella nipponica*** Franch. et Sav., KT161786, KT161542. ***Selaginella ornata*** Spring, CHINA, Guizhou, *X.–C. Zhang et al. 7082* (PE), KY068360*, KY068355*. ***Selaginella pallidissima*** Spring, KT161796, KT161556. ***Selaginella pennata*** Spring, KT161798, KT161558. ***Selaginella picta*** A. Braun ex Baker, KT161800, KT161561. ***Selaginella pulvinata*** (Hook. et Grev.) Maxim., KT161811, KT161576. ***Selaginella remotifolia*** Spring, KT161814, KT161580. ***Selaginella repanda*** (Desv.) Spring, KT161816, KT161582. ***Selaginella roxburghii*** (Hook. & Grev.) Spring, –, EU140945. ***Selaginella sanguinolenta*** (L.) Spring, KT161822, KT161589. ***Selaginella scabrifolia*** Ching & C.H.Wang, KT161824, KT161593. ***Selaginella selaginoides*** (L.) Link, AF419000, AF419048. ***Selaginella sibirica*** (Milde) Hieron., AF419032, AF419076. ***Selaginella superba*** Alston, KT161842, KT161616. ***Selaginella tamariscina*** (P.Beauv.) Spring, –, AB574655. ***Selaginella trachyphylla*** A.Braun ex Hieron., –, KT161620. ***Selaginella vardei*** Levl., KT161853, KT161628.
